# Supplementary material for: Identification of photoperiod-induced specific miRNAs in the adrenal glands of Sunite sheep (Ovis aries)
Source: Front Vet Sci. 2022 Jul 22;9:888207. doi: 10.3389/fvets.2022.888207 (PMC9354845; doi:10.3389/fvets.2022.888207)
Supplement: Supplementary file 1 [file Data_Sheet_1.ZIP › Supplementary file/miRDeep2 file.zip!/miRDeep2 file/Novel_Structure/SP42A1/Novel_708.pdf]

Provisional ID	: NC_019460.2_6118
Score total	: 0.3
Score for star read(s)	: -1.3
Score for read counts	: -1.3
Score for mfe	: 1.4
Score for randfold	: 1.6
Score for cons. seed	:
Total read count	: 9
Mature read count	: 9
Loop read count	: 0
Star read count	: 0

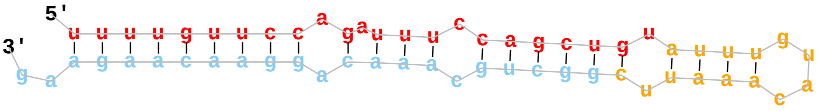

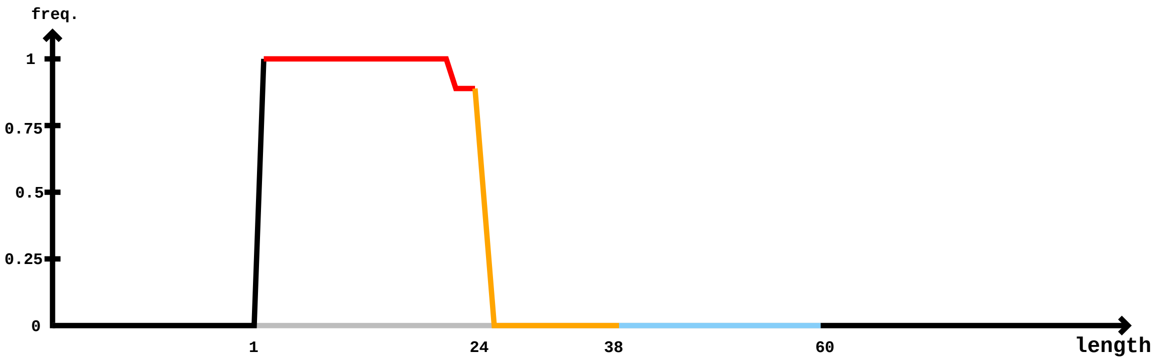

Mature **Star**

```
5'- accuuuggaguuggaugacuuuuuguuccagauuuccagcuguauuuguacaaaauucggcugcaaacaggaacaagaaguugucgcuuaacucaaguagauccuuggaac   -3'    exp
    ..((...((((((((((((((((((((((.(.(((.((((((.((((....)))).)))))).)))).))))))))))))))))...))))))....))............    reads     mm     sample
    ....................uuuuguuccagauuuccagc.......................................................................    1         0      seq
    ....................uuuuguuccagauuuccagcugu....................................................................    5         0      seq
    ....................uuuuguuccagauuuccagcugua...................................................................    3         0      seq
```
